# NOD1, NOD2, PYDC1, and PYDC2 gene polymorphisms in ovarian endometriosis

**DOI:** 10.3389/fmed.2024.1495002

**Published:** 2025-02-17

**Authors:** Hakan Kula, Beste Balbal, Tunc Timur, Pelin Yalcın, Onur Yavuz, Sefa Kızıldag, Emine Cagnur Ulukus, Cemal Posaci

**Affiliations:** ^1^Department of Obstetrics and Gynecology, Dokuz Eylul University School of Medicine, İzmir, Türkiye; ^2^Department of Medical Biology, Dokuz Eylul University, İzmir, Türkiye; ^3^Department of Pathology, Dokuz Eylul University School of Medicine, İzmir, Türkiye

**Keywords:** endometriosis, infertility, pain, gene polymorphism, NOD, PYDC

## Abstract

**Background:**

Endometriosis, a prevalent chronic gynecologic disorder, significantly impacts women’s health, with both genetic and environmental factors contributing to its heritability. Within the adaptive immune system, the NOD-like receptors (NLR) pathway plays pivotal roles in various autoinflammatory diseases, regulating interleukins, proinflammatory cytokines, and NF-κB activity. However, the potential association between single nucleotide polymorphisms (SNPs) of the NOD1, NOD2, PYDC1, and PYDC2 genes and the predisposition to endometriosis risk remains unexplored.

**Methods:**

In this cross-sectional study, 54 patients diagnosed with ovarian endometriosis and 54 control subjects were included. The genetic SNPs of NOD1 (rs2075820 and rs2075818) and NOD2 (rs104895461) were assessed using the PCR-RFLP (polymerase chain reaction-restriction fragment length polymorphism) method. Additionally, the polymorphisms of PYDC1 and PYDC2 were evaluated using Sanger sequencing. After conducting polymorphism analysis, the genetic profiles were assessed with the clinical manifestations and the size of ovarian endometriomas, categorized as either small (<4 cm) or large (≥4 cm).

**Results:**

Significant differences in the NOD1 rs2075820 (G: A) genotypes were found. The GG genotype was more prevalent in endometriosis patients (*p* = 0.04), while the GA genotype was less common (*p* = 0.029). The AA genotype was associated with higher rates of perimenstrual gastrointestinal symptoms (*p* = 0.005) and infertility (*p* = 0.037). The PYDC2 rs293833 variant was detected in 22.2% of patients. Carriers of this variant exhibited higher rates of perimenstrual gastrointestinal symptoms (*p* = 0.004), infertility (*p* = 0.001) and larger endometriomas (≥4 cm) (*p* < 0.001). No significant differences were found in NOD1 rs2075818 genotypes (*p* = 0.89) and no polymorphisms were detected in NOD2 or PYDC1 genes.

**Conclusion:**

These findings emphasize the influence of genetic polymorphisms on the clinical manifestations of endometriosis. Specifically, gene polymorphisms in NLRs have been found to significantly impact infertility and increase endometrioma size.

## Introduction

Endometriosis is characterized by an estrogen-dependent chronic inflammatory pathology that affects reproductive-aged women with pelvic pain and infertility (1). Understanding the mechanisms underlying endometriosis is crucial due to its clinical and therapeutic relevance. While numerous theories have been proposed, none fully explain the disease’s progression and diverse clinical manifestations. Sampson’s retrograde menstruation theory remains the most widely cited explanation (2). However, this theory does not adequately explain why only 10% of women with retrograde menstrual flow develop endometriosis.

A common element in all theories is the dysregulation of hormonal signaling and an inflammatory microenvironment, which, together with genetic and epigenetic factors, drive the disease’s initiation, persistence, and progression (3). Genetic predisposition is significant, as daughters of affected mothers have double the risk of developing endometriosis, and monozygotic twins show a 51% increased risk (4, 5). Ovarian endometriomas are a significant and prominent component of endometriosis. About 17–44% of patients with endometriosis have endometriomas, with bilateral endometriomas occurring in 19–28% of these patients (6). Endometriosis is a chronic pelvic inflammatory condition where local inflammation significantly contributes to pain and infertility. Excessive reactive oxygen species (ROS) production affects gene expression, with NF-κB involvement in the disease. Activated NF-κB in lesions and macrophages drives proinflammatory cytokine production, supporting lesion formation and persistence (7).

The innate immune system detects various danger and pathogen-associated molecular patterns through pattern-recognition receptors (PRRs), such as nod-like receptors (NLRs) (8). The NLR family comprises over 20 members, including nucleotide-binding oligomerization domain-containing proteins 1 and 2 (NOD1 and NOD2) (9). Engagement of NLRs triggers cooperative signaling between mitogen-activated protein kinase (MAPK) and nuclear factor kappa B (NF-κB) pathways, leading to the transcription of pro-inflammatory cytokines, assembly of NLR inflammasomes, and cell death (10). Moreover, pyrin-only protein/pyrin domain (POP/PYDC) domain proteins also disrupt NF-κB signaling by forming an inflammasome complex by certain NLRs and interleukins (11). Studies highlight that mutations and dysregulation in NLRs, such as NOD2 and NLRP3, significantly impact these pathways, altering immune responses and contributing to diseases like Crohn’s disease and cryopyrinopathies. Polymorphisms in the NOD1 and NOD2 genes can disrupt the balance between pro- and anti-inflammatory cytokines, fostering chronic inflammation and increasing the risk of cancer. These findings emphasize the critical role of structure–function relationships in understanding NLR-mediated immune regulation and their relevance to disease pathogenesis (12).

Polymorphisms play a crucial role in understanding the genetic underpinnings of complex diseases, including endometriosis. Given the multifactorial nature of endometriosis, the identification of genetic variants that contribute to disease susceptibility has significant implications for advancing diagnostic and therapeutic strategies. However, there remains a substantial research gap in understanding the precise contribution of genetic polymorphisms to endometriosis, with many studies producing inconsistent results across populations and ethnic groups. This variability underscores the complexity of genetic influence on endometriosis, suggesting that multiple, potentially interacting loci may contribute to its pathology (13, 14).

In this study, we aim to investigate inflammasome regulators PYDC1 and PYDC2 and genetic variations in the NOD1 and NOD2 genes in patients with ovarian endometriosis. Additionally, we will evaluate the genetic profile of these patients with the size of the endometriomas and their clinical symptoms.

## Method

### Subjects

All subjects provided written informed consent for inclusion before participating in the study. The study was conducted by the Declaration of Helsinki of 1975 (as revised in 2013), and the protocol was reviewed and approved by the Health Research Ethics Committee of Dokuz Eylul University (7511-GOA). Blood samples were collected from a total of 108 patients who had either undergone laparoscopic surgery or exploratory laparotomy between March 2022 and November 2023. The study population comprised 54 patients diagnosed with ovarian endometriosis (endometriosis group) and 54 control subjects without endometriosis (control group).

#### Endometriosis group

Diagnosis of endometriosis was confirmed and classified based on visual and histopathological examinations according to the American Association of Gynecologic Laparoscopists (AAGL) and the revised American Society for Reproductive Medicine (rASRM) Endometriosis Classification Systems (15, 16). According to AAGL Classification, 16 (29.6%) were in stage II, 34 (63%) in stage III, and 4 (7.4%) in stage IV. When classified with rASRM, 44 patients (81.5%) were classified as stage 3, and 10 patients (18.5%) as stage 4. To explore potential genetic differences related to endometrioma size, a subgroup analysis was performed, categorizing endometriomas as larger (≥4 cm) or smaller (<4 cm).

The control group consisted of patients who underwent surgery for fibroids, menorrhagia, benign adnexal masses, and pelvic organ prolapse. Endometriosis was ruled out in these patients through histopathological evaluation. Patients with additional autoimmune diseases, pelvic inflammatory disease, or gynecological malignancies were excluded from both the endometriosis and control groups.

### Genotyping polymorphisms

**Figure 1:**
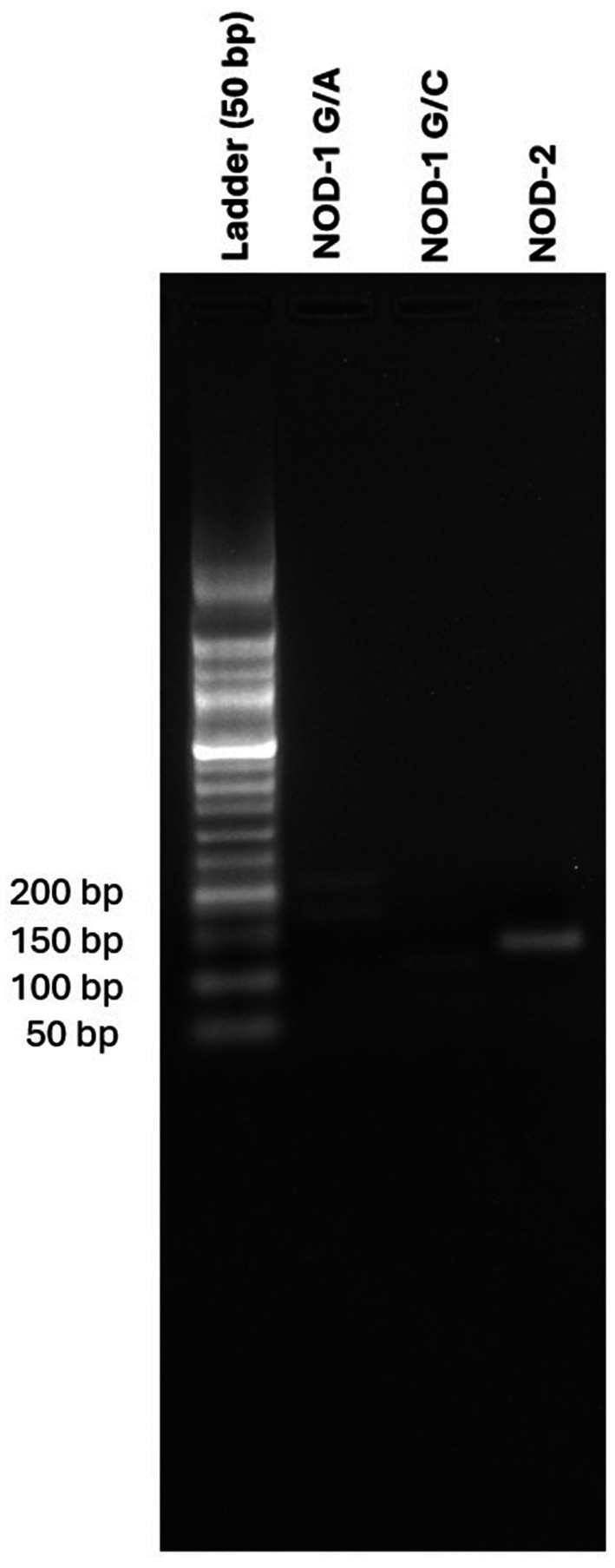
Comparative gel electrophoresis of allele separation with DNA ladder for NOD1 G/A, NOD 1 G/C, and NOD2 variants.

DNA was collected in 5 mL peripheral blood, followed by ficol separation (Sigma Histopaque-1077, cat no: 10771). DNA isolation was then performed using Trizol (Invitrogen TM, cat no: 15596018). Conventional polymerase chain reaction (PCR) was conducted using Taq DNA Polymerase (A.B.T., cat no: E02-01-50) for the target genes, with the following protocol: 40 cycles of denaturation at 95°C for 10 s, annealing at 60°C for 30 s, and extension at 65°C for 95 min. Primer sequences for the target genes previously created before (17). Genotypes rs2075818 and rs104895461 were determined using the PCR-restriction fragment length polymorphism (PCR-RFLP) method. PCR products were incubated overnight at 37°C with specific restriction enzymes for the restriction enzyme process. Samples were loaded onto a 2% agarose gel to determine allele separation and visualized (Figure 1).

The PCR primers used for Sanger sequencing of the PYDC1 and PYDC2 genes are listed in Supplementary material 1. After PCR amplification, the products were purified, and sequencing reactions were performed (Macrogen Europe). After completing the electrophoresis process, the samples were analyzed using the “Sequence Analysis” program. Sequence comparisons and analyses were conducted using the MutationSurveyor 1.2 program.

### Statistical analysis

The sample size was calculated using a power analysis, achieving 95% confidence level. Hardy–Weinberg equilibrium was assessed for each evaluated SNP. The student’s t-test was employed to compare means of continuous variables. The Chi-square test was used to compare mutations and allele frequencies among groups and clinical features within subgroups categorized by endometrioma size. Statistical analyses were conducted using IBM SPSS version 26.0, with a *p*-value of <0.05 accepted as statistically significant.

## Results

There were no significant differences between endometriosis patients and control subjects regarding age (39.28 ± 8.22 vs. 39.31 ± 7.86), BMI (23.1 ± 1.4 vs. 22.8 ± 1.5), and age at menarche (12.4 ± 1.6 vs. 12.1 ± 1.8) (*p* > 0.05). Out of the patients studied, 54 had ovarian endometriosis. Of these, 42 patients (77.8%) had unilateral ovarian endometriosis, while 12 patients (22.2%) had bilateral involvement. In the endometriosis group, 48 patients (88.9%) underwent first-time surgery, and 6 patients (11.1%) had recurrent endometriomas.

The symptoms reported by patients with endometriosis included dysmenorrhea 38 (70.4%), dyspareunia 28 (51.9%), perimenstrual gastrointestinal system (GIS) complaints 23 (42.6%), ovulatory pain 18 (33.3%), menorrhagia 18 (33.3%), perimenstrual genitourinary system (GUS) complaints 10 (18.5%). Infertility was present in 13 patients (24.1%), with 9 patients (16.7%) experiencing primary infertility and 4 patients (7.4%) experiencing secondary infertility (Table 1).

**Table 1:** Characteristics of ovarian endometriosis patients.

Characteristics of ovarian endometriosis group	*n* (%)
Unilateral Endometrioma	42 (77.8)
Bilateral Endometrioma	12 (22.2)
AAGL	
Stage 2	16 (29.6)
Stage 3	34 (63)
Stage 4	4 (7.4)
rASRM	
Stage 3	44 (81.5)
Stage 4	10 (18.5)
First-time endometriosis surgery	48 (88.9)
Recurrent endometriosis surgery	6 (11.1)
Medical treatment	
NSAIDs	39 (72.2)
OCPs	20 (37.0)
Oral Progestins	16 (29.6)
GnRH Agonists	2 (3.7)
LNG-IUD	4 (7.4)
Clinical complaints	
Dysmenorrhea	38 (70.4)
Ovulatory pain	18 (33.3)
Menorrhagia	18 (33.3)
Dyspareunia	28 (51.9)
Perimenstrual GIS complaints	23 (42.6)
Perimenstrual GUS complaints	10 (18.5)
Infertility	13 (24.1)
Primary infertility	9 (16.7)
Secondary infertility	4 (7.4)

AAGL, the American Association of Gynecologic Laparoscopists; rASRM, the revised American Society for Reproductive Medicine; NSAIDs, Nonsteroidal Anti-inflammatory Drugs; OCPs, Combined Oral Contraceptive Pills; LNG-IUD, Levonorgestrel-releasing intrauterine system; GIS, Gastrointestinal; GUS, Genitourinary. ^*^This table provides a comprehensive overview of the characteristics observed in patients with ovarian endometriosis, including the distribution of unilateral and bilateral endometriomas, surgical stages, treatment history, and clinical complaints.

There were no significant differences in allele frequencies between endometriosis and control subjects for NOD1 rs2075820 (G vs. A) (*p* = 0.89) and rs2075818 (G vs. C) (*p* = 0.89). A statistically significant difference in the distribution of the rs2075820 (NOD1 G/A) genotypes was observed between endometriosis patients and control subjects. The GG wild-type genotype was found to be significantly more prevalent in the endometriosis group 17 (31.5%) compared to the control group 11 (20.3%) (*p* = 0.04). Conversely, the GA genotype was significantly less common among endometriosis patients 28 (51.9%) than in controls 39 (72.2%) (*p* = 0.029). Although the AA genotype was more frequent in endometriosis patients 9 (16.6%) than in control subjects 4 (7.5%), this difference did not reach statistical significance (*p* = 0.13) (Table 2).

**Table 2:** NOD1 (rs2075820 and rs2075818) allele frequencies and genotypes.

	Endometriosis	Control	*p*
	*n* (%)	*n* (%)	
NOD 1 (rs2075820)			
Allele frequencies			
G	62 (57.4)	61 (56.4)	0.89
A	46 (42.6)	47 (43.6)	
Genotype frequencies			
GG	17 (31.5)	11 (20.3)	**0.04**
GA	28 (51.9)	39 (72.2)	**0.029**
AA	9 (16.6)	4 (7.5)	0.13
NOD 1 (rs2075818)			
Allele frequencies			
G	51 (47.2)	53 (49)	0.89
C	57 (52.8)	55 (51)	
Genotype frequencies			
GG	7 (13)	7 (13)	0.54
GC	37 (68.5)	39 (72.2)	0.67
CC	10 (18.5)	8 (14.8)	0.60

A, Adenine; G, Guanine; C, Cytosine. *The Chi-square test. **An allele refers to a variant form of a gene. In this context, each individual has two alleles for each gene—one inherited from each parent. A genotype refers to the combination of alleles an individual possesses for a particular gene. For a gene with two possible alleles (like G and A or G and C), the possible genotypes are: Homozygous for one allele (GG, AA, or CC). Heterozygous (GA and GC). Bold values represent statistically significant results (*p* < 0.05).

No significant differences were detected when evaluating the NOD1 (rs2075818) genotypes between endometriosis patients and control subjects. The frequencies of the GG genotype were identical in both groups (13% vs. 13%; *p* = 0.54). Similarly, the distribution of the GC genotype (68.5% in endometriosis patients vs. 72.2% in controls; *p* = 0.67) and the CC genotype (18.5% in endometriosis patients vs. 14.8% in controls; *p* = 0.6) showed no significant differences (Table 2). No polymorphisms were detected at the NOD2 (rs104895461) and PYDC1 genes. PYDC2 rs293833 (c.242A>G) variant was detected in 12 endometriosis patients (22.2%).

We also evaluated the association of three polymorphisms in the NOD1, NOD2, and PYDC2 genes with the clinical manifestations of endometriosis. The NOD1 rs2075820 AA genotype was associated with significantly higher rates of perimenstrual GIS symptoms 8 (88.9%) compared to other NOD1 rs2075820 genotypes 17 (37.8%) (*p* = 0.005). Additionally, infertility was significantly more common in patients with the AA genotype 5 (55.5%) compared to those with other genotypes 8 (17.8%) (*p* = 0.037) (Table 3).

**Table 3:** NOD1 rs2075820 gene polymorphism analysis according to the recessive model.

	GG + GA	AA	*p*
	*n* %	*n* %	
Unilateral endometrioma	35 (77.8)	7 (77.8)	0.99
Bilateral endometrioma	10 (22.2)	2 (22.2)	0.99
Dysmenorrhea	34 (75.6)	4 (44.4)	0.06
Ovulatory Pain	17 (37.8)	1 (11.1)	0.12
Menorrhagia	16 (35.6)	2 (22.2)	0.43
Dyspareunia	24 (53.3)	4 (44.4)	0.62
Perimenstrual GIS symptoms	17 (37.8)	8 (88.9)	**0.005**
Perimenstrual GUS symptoms	9 (20.0)	1 (11.1)	0.53
Infertility	8 (17.8)	5 (55.5)	**0.037**
Endometrioma size			
Small endometrioma (<4 cm)	12 (26.7)	5 (55.5)	0.08
Large endometrioma (≥4 cm)	33 (73.3)	4 (44.5)	

GIS, Gastrointestinal; GUS, Genitourinary. *The Chi-square tests. **This table provides a comparison of clinical characteristics and endometrioma sizes in patients with different genotypes (GG + GA vs. AA) for a specific polymorphism, with *p*-values indicating statistical significance for each comparison. Additionally, analysis for the G and C polymorphism (GG + GC vs. CC) was not included due to the absence of significant results. Bold values represent statistically significant results (*p* < 0.05).

PYDC2 rs293833 (c.242A>G) positive patients exhibited a lower incidence of dysmenorrhea compared to negative patients (41.7% vs. 78.6%; *p* = 0.014). Moreover, perimenstrual gastrointestinal symptoms were significantly more prevalent in positive patients (83.3% vs. 35.7%; *p* = 0.004). Additionally, PYDC2-positive patients had significant differences in infertility and the presence of larger endometriomas. Infertility rates were markedly higher in positive patients (66.6% vs. 11.9%; *p* = 0.001), and large endometriomas were more frequently observed in positive patients (90.9% vs. 62%; *p* < 0.001) (Table 4).

**Table 4:** PYDC2 gene polymorphism analysis for endometriosis patients.

	PYDC2 rs293833 (c.242A > G)		
	Positive	Negative	*p*
	*n* (%)	*n* (%)	
Unilateral endometrioma	9 (75)	33 (78.6)	0.79
Bilateral endometrioma	3 (25)	9 (21.4)	0.79
Dysmenorrhea	5 (41.7)	33 (78.6)	**0.014**
Ovulatory Pain	6 (50)	12 (28.6)	0.16
Menorrhagia	2 (16.7)	16 (38.1)	0.16
Dyspareunia	7 (58.3)	21 (50)	0.61
Perimenstrual GIS symptoms	10 (83.3)	15 (35.7)	**0.004**
Perimenstrual GUS symptoms	2 (16.7)	8 (19)	0.85
Infertility	8 (66.6)	5 (11.9)	**0.001**
Endometrioma size			
Small endometrioma (<4 cm)	1 (9.1)	16 (38)	
Large endometrioma (≥4 cm)	11 (90.9)	26 (62)	**<0.001**

GIS, Gastrointestinal; GUS, Genitourinary. ^*^The Chi-square test. ^**^This table compares clinical characteristics and endometrioma sizes between patients with positive and negative PYDC2 rs293833 (c.242A > G) polymorphism, with associated *p*-values indicating statistical significance. Bold values represent statistically significant results (*p* < 0.05).

## Discussion

Genome-wide association studies (GWAS) have described that ovarian endometriosis partly contributes to the larger effect sizes observed in ASRM Stage 3-4, indicating a genetic basis distinct from other disease manifestations (18). In this study, we hypothesized that genetic factors may play a role in the pathophysiology of ovarian endometriosis. This study aimed to assess the genetic predisposition to the development and characteristics of this disease, focusing on the presence of four specific inflammasome-related polymorphisms: NOD1 (rs2075820 and rs2075818), NOD2 (rs104895461), PYDC1, and PYDC2 gene polymorphisms. This is the first report to detail the analysis of gene polymorphisms for these genes in endometriosis.

Previously, NOD1 and NOD2 genes were assessed for their potential predisposition to endometrial cancer; however, no associations were observed (19). Our study revealed that the NOD1 rs2075820 had lower (G>A) genotypes in endometriosis patients when compared with the control group. A pro-apoptotic protein NOD1 can trigger apoptosis through interactions with the caspase pathway whereas NF-κB serves to suppress the apoptotic process (20). NOD proteins can initiate signaling pathways involving both NF-κB and caspase in endometriosis. On the other hand, the allele frequencies of G and A in NOD1 rs2075820 did not differ significantly. Other studies revealed that the presence of the A allele of rs2075820 correlated with decreased expression and activation of NF-κB when intracellular *Propionibacterium acnes* (*P. acnes*) infection present in the Japanese population (21).

A few studies investigated the expression of NODs in the female reproductive tract. NOD1 and NOD2 are differentially expressed and regulated in the human endometrium, playing roles in the innate immune response and potentially in the inflammatory events associated with menstruation with interleukins (22). In another study, ectopic endometrial stromal cells showed increased levels of NOD1 expression and interleukin-8, while the NOD1 inhibitor ML-130 suppressed proliferation, clonal expansion, invasion, and migration of these cells without impacting apoptosis (23).The pathophysiological mechanism behind diminished ovarian reserve in endometriosis remains unclear. It is debated whether endometriomas reduce functional tissue through mechanical stretching (space-occupying effect) or direct inflammatory impact. Ovarian endometriomas contain immune components like reactive oxygen species (ROS), metalloproteinases, and cytokines, which may progressively damage the ovarian stroma and reduce the primordial follicular reserve over time (24).

Ovarian endometriosis poses a challenge to ovarian reserve, though the extent of its uniform impact on reserve remains debated. A retrospective study on women with ovarian endometriomas (mean diameter 26 ± 8 mm) undergoing multiple ovarian stimulation cycles found consistent oocyte retrieval rates from affected ovaries across cycles, at 44% for both initial and subsequent cycles. Another study reported a statistically significant 26% decrease in anti-müllerian hormone (AMH) levels over six months in 40 women with endometriomas (mean diameter 46 ± 17 mm), indicating a progressive decline in ovarian reserve (25).

Ovarian endometrioma size has been studied in relation to ovarian stimulation, with a 4 cm diameter threshold commonly used to indicate potential impact on ovarian response. Generally, small cysts have minimal effects, while larger cysts can significantly affect ovarian function. Our findings reveal that the NOD1 rs2075820 AA phenotype and PYDC2 rs293833 (c.242A > G) polymorphism are strongly associated with female infertility. Additionally, PYDC2 rs293833 (c.242A > G) correlates with larger endometriomas (≥4 cm). Subgroup analysis supports GWAS recommendations for assessing genetic variations, particularly in cases with larger ovarian cysts and severe endometriosis, to improve reproductive outcomes.

The primary treatments for endometriosis include surgery and pharmacological options like hormone therapy and NSAIDs for pain management. Surgical excision can improve symptoms and fertility; however, recent reviews show recurrence rates of 21.5% at 2 years and 40–50% at 5 years, indicating that recurrences and repeat surgeries may exacerbate pain and further reduce fertility (26).

Therefore, regular and long-term medication use is recommended to prevent postoperative recurrence of endometriosis. However, hormone therapies, due to estrogen’s role in endometriosis development, may suppress follicular development and ovulation, making treatment challenging for women seeking pregnancy. NLRs are hypothesized as promising therapeutic targets for addressing inflammation-associated endometriosis via their pivotal role in innate immunity (10). NLR family pyrin domain containing 3 (NLRP3) and NLR family CARD domain containing 5(NLRC5) have prominent improving effects on endometriosis with altering fibrosis and inflammation in previous studies (27, 28).

The NLRP3/IL-1β pathway plays a role in endometriosis development, and NLRP3 inhibitors may help reduce ovarian endometrioma size and improve ovarian function (29). In a study, increased NOD1 expression and inflammatory cytokines in ectopic endometrial cells in peritoneal fluid, with the NOD1 inhibitor ML130 significantly reducing cell viability and cytokine production (30). Furthermore, mifepristone has been shown to exhibit protective effects against NLRP1 inflammasome activation and to minimize damage to hippocampal neurons caused by dexamethasone (31). Thus, strategies targeting the inflammasome axis may serve as potential therapeutic options for treating endometriosis.

Women with pelvic endometriosis often experience pain due to pelvic visceral hypersensitivity, along with abdominal and pelvic discomfort. Studies show that the inflammatory microenvironment within ectopic lesions activates sensory nerve endings through inflammatory mediators, amplifying pain signal transmission (32). This hypothesis is reinforced by fluctuations in cyclic inflammatory markers during the menstrual cycle, which correlate with heightened gastrointestinal symptoms. The overlap between endometriosis and irritable bowel syndrome (IBS)—more commonly diagnosed in women with pelvic endometriosis—adds complexity to interpreting gastrointestinal symptoms. Additionally, endometriosis patients show lower pain thresholds in response to bowel distension and other gastrointestinal triggers (33, 34). In another study, NOD1 rs2075820 was not associated with inflammatory bowel disease in the Turkish population (35).

Our findings suggest that NOD1 rs2075820 AA phenotype and PYDC2 rs293833 (c.242A>G) polymorphism is strongly associated with increased gastrointestinal complaints in ovarian endometriosis patients. The localization of ovarian endometriosis in areas closely related to the terminal parts of the colon, along with its inflammatory characteristics and local factors such as prostaglandin release, may explain the increased incidence of gastrointestinal complaints in endometriosis patients. However, painful symptoms associated with deep infiltrative endometriosis (DIE) may also cause pain characteristics, often specific to precise anatomical locations or affected organs, such as severe deep dyspareunia or painful defecation.A limitation of the study includes the potential for more robust results if the sample size for subgroup analysis is increased, even though the sample size was previously calculated specifically for ovarian endometriosis. On the other hand, to the best of our knowledge, this is the first study to evaluate endometriomas with their sizes and genetic profiles together. Obtaining significant differences between these groups may provide valuable insights for further studies.

## Conclusion

Our study shows a correlation between genetic predispositions, inflammatory pathways, and the clinical manifestations of ovarian endometriosis. By investigating specific inflammasome-related polymorphisms, NOD1, and PYDC2 gene variants, we have uncovered potential associations with infertility and gastrointestinal complaints in affected individuals. These findings imply that the inflammatory microenvironment substantially influences infertility, particularly through pathways associated with the inflammasome complexes. The importance of considering genetic variations is shown in the evaluation and management of endometriosis, especially in subgroups characterized by severe disease phenotypes. Moreover, our results highlight the complex nature of endometriosis pathophysiology, implicating not only mechanical and inflammatory processes but also genetic factors in disease progression and symptomatology.

## Data Availability

The original contributions presented in the study are included in the article/Supplementary material. Further inquiries can be directed to the corresponding author.
