# Supplementary material for: NOD1, NOD2, PYDC1, and PYDC2 gene polymorphisms in ovarian endometriosis
Source: Front Med (Lausanne). 2025 Feb 17;11:1495002. doi: 10.3389/fmed.2024.1495002 (PMC11872704; doi:10.3389/fmed.2024.1495002)
Supplement: Supplementary file 1 [file Supplementary_file_1.docx]

**SUPPLEMENTS**

| **Reference ID** | **Primer sequence** | **Annealing temperatures (°C)** | **Restriction enzymes** | **Cleavage temperatures (°C)** | **Base pair** | **Fragment lenght differences for RFLP** |
| --- | --- | --- | --- | --- | --- | --- |
| **rs2075820 (NOD1 G/A)** | **F: 5’-TGAGACCATCTTCATCCTGG-3’**  **R: 5’-CTTCCCACTGAGCAGGTTG-3’** | **57** | **AvaI** | **37** | **375 bp** | **207/168** |
| **rs2075818 (NOD1 G/C)** | **F: 5’-GCAATCGGGAACTTCTGGTCACT-3’**  **R: 5’-GGGGCAGGCACACACAATCTC-3’** | **58** | **HaeIII (BsuRI)** | **37** | **113 bp** | **81/32 bp** |
| **rs104895461 (NOD2 R334Q/R334W)** | **F: 5’-TGACGATGCGGACACTGTGC-3’**  **R: 5’ AACAGGACACGGTCAGGGTG 3’** | **57** | **MspI** | **37** | **261 bp** | **134/127 bp** |
| **PYDC1** | **F:5’-GTGGCAGAATCACAACTCGAACTC-3’**  **R: 5’-ACCTGACGCGTGGGTCCTT-3’** | **60** | **-** | **-** | **-** | **-** |
| **PYDC2** | **F: 5’-AGAACCAGAGCATAGGGCCAG-3’**  **R:5’-GCAGAACCAAGGCTAAAGCAACATC-3’** | **60** | **-** | **-** | **-** | **-** |

**Table 1. Primer Details and Fragment Length Differences**

| **Reference ID** | **Gene** | **Chromosome Number** | **Altered Nucleotide** |
| --- | --- | --- | --- |
| **rs2075820** | **NOD1** | **chr7:30452621 (GRCh38.p14)** | **c.796G>A**  **c.652G>A**  **c.52G>A** |
| **rs2075818** | **NOD1** | **chr7:30456766 (GRCh38.p14)** | **c.156C>T**  **c.156C>G**  **c.156C>A** |
| **rs293833** | **PYDC2** | **chr3:191461404 (GRCh38.p14)** | **c.242A>G** |

**Table 2: Genetic Variants of NOD1 and PYDC2: Chromosomal Locations and Nucleotide Alterations**
